# The methylome of the gut microbiome: disparate Dam methylation patterns in intestinal *Bacteroides dorei*

**DOI:** 10.3389/fmicb.2014.00361

**Published:** 2014-07-17

**Authors:** Michael T. Leonard, Austin G. Davis-Richardson, Alexandria N. Ardissone, Kaisa M. Kemppainen, Jennifer C. Drew, Jorma Ilonen, Mikael Knip, Olli Simell, Jorma Toppari, Riitta Veijola, Heikki Hyöty, Eric W. Triplett

**Affiliations:** ^1^Department of Microbiology and Cell Science, Institute of Food and Agricultural Sciences, University of FloridaGainesville, FL, USA; ^2^Department of Clinical Microbiology, University of Eastern FinlandKuopio, Finland; ^3^Immunogenetics Laboratory, University of TurkuTurku, Finland; ^4^Children's Hospital, University of Helsinki and Helsinki University Central HospitalHelsinki, Finland; ^5^Diabetes and Obesity Research Program, University of HelsinkiHelsinki, Finland; ^6^Department of Pediatrics, Tampere University HospitalTampere, Finland; ^7^Department of Pediatrics, Turku University Hospital, University of TurkuTurku, Finland; ^8^Department of Pediatrics, Oulu University Hospital, University of OuluOulu, Finland; ^9^School of Medicine, University of TampereTampere, Finland

**Keywords:** DNA adenine methyltransferase, GATC, metagenomics, genomics, epigenetics

## Abstract

Despite the large interest in the human microbiome in recent years, there are no reports of bacterial DNA methylation in the microbiome. Here metagenomic sequencing using the Pacific Biosciences platform allowed for rapid identification of bacterial GATC methylation status of a bacterial species in human stool samples. For this work, two stool samples were chosen that were dominated by a single species, *Bacteroides dorei*. Based on 16S rRNA analysis, this species represented over 45% of the bacteria present in these two samples. The *B. dorei* genome sequence from these samples was determined and the GATC methylation sites mapped. The *Bacteroides dorei* genome from one subject lacked any GATC methylation and lacked the DNA adenine methyltransferase genes. In contrast, *B. dorei* from another subject contained 20,551 methylated GATC sites. Of the 4970 open reading frames identified in the GATC methylated *B. dorei* genome, 3184 genes were methylated as well as 1735 GATC methylations in intergenic regions. These results suggest that DNA methylation patterns are important to consider in multi-omic analyses of microbiome samples seeking to discover the diversity of bacterial functions and may differ between disease states.

## Introduction

DNA adenosine methylation (Dam methylation) of the 5′-GATC-3′ motif in bacterial genomes, particularly in *Escherichia coli*, has been well characterized for decades (Marinus, 1987; Barras and Marinus, 1989; Marinus and Casadesus, 2009). Of all of the DNA methylation systems known in bacteria, GATC methylation appears to have the highest impact on gene expression (Barras and Marinus, 1989). The biochemistry of the adenosine methylation reaction catalyzed by bacterial DNA adenine methyltransferase (DamMT) is also well characterized (Thielking et al., 1997; Urig et al., 2002). As DamMT mutants are not lethal, the phenotype of DamMT mutants in several bacteria have shown that methylation of the GATC motif affects gene expression of many processes including chromosome replication, mismatch repair, and nucleoid structure (Løbner-Olesen et al., 2005). The GATC methylation system has been shown to increase virulence in *Salmonella enterica* serovar Typhimurium, *Vibrio cholerae*, *Yersinia pestis*, and *Yersinia pseudotuberculosis* (Heithoff et al., 1999; Julio et al., 2001, 2002; Robinson et al., 2005). As a result, significant efforts have been made in recent years to design antibiotics that inhibit DamMT (Mashhoon et al., 2004, 2006; Hobley et al., 2012; McKelvie et al., 2013).

Despite the enormous interest in recent years to characterize the bacterial diversity of the human microbiome, particularly as it relates to gut diseases, the role that DNA methylation plays in function of the microbiome remains unknown. The PacBio RS II system is capable of detecting DNA methylation through analysis of polymerase kinetics (Flusberg et al., 2010; Clark et al., 2012; Fang et al., 2012; Murray et al., 2012). This technology was used here to discover the profound differences in the extent of Dam methylation in one dominant bacterial species in the gut between two children at high genetic risk for type 1 diabetes and 1 year prior to the development of type 1 diabetes autoimmunity in one of these children.

## Materials and methods

The stool samples used here were collected by the Finnish Type 1 Diabetes Prediction and Prevention Study (DIPP) (Kukko et al., 2005). Newborns were screened for high-risk HLA-DR and HLA-DQ genotypes using a previously described method (Kukko et al., 2005). Stool samples were collected by the subjects' parents at home and mailed to the DIPP Virus Laboratory for virology in Tampere, Finland, where they were stored at −80°C. Detection of beta-cell autoimmunity was done as described in Parikka et al. (2012). Sample 105 was collected from the subject at 13.5 months of age. This subject became autoimmune for type 1 diabetes at 15.1 months of age. Sample 439 was collected from a subject who remained healthy at 3.3 months of age. Both subjects were genetically at high risk for type 1 diabetes given their HLA genotype.

In this study, DNA extraction and 16S rRNA amplification, sequencing, and analysis was done as described previously (Fagen et al., 2012) except the Qiagen AllPrep DNA/RNA/Protein Mini Kit (QIAGEN) was used to extract DNA, RNA, and protein from stool. Based on the 16S rRNA results, two samples were chosen for long-read Pacific Biosciences sequencing based on the high relative abundance of this organism was 63.7 and 47.9% from samples 105 and 439, respectively. These samples, 105 and 439, were collected from children who became autoimmune or remained healthy, respectively.

Pacific Biosciences (PacBio RS II system) library construction and sequencing was done by the University of Florida's Interdisciplinary Center for Biotechnology Research. Prior to sequencing, a PacBio library was made with SMRTbell Adaptors. The *Bacteroides dorei* genome was assembled to closure from sample 105 after obtaining eight SMRT cells of sequence data. A total of 1,502,920 reads and 1,860,712,096 bases were obtained, with a mean read length of 2706 bp. Average read quality was 0.848. The initial Pacbio reads were error corrected using the Pacbio RS_PreAssembler.1 module (Koren et al., 2012) with minimum subread length of 400 bp, minimum read quality 0.60, and minimum seed read length of 3800 bp. The error correction process yielded 47,654 reads of 2378 bp average length. Reads were binned according to coverage reported by the Pacbio RS_PreAssembler.1 protocol, filtering out reads with lower than 200× coverage. A set of 27 contigs was assembled directly from the binned reads using SPAdes assembler v3.0 (Bankevich et al., 2012). A single scaffold was obtained by detecting overlaps with Mauve 2.3.1 (Darling et al., 2010) and manually assembling the remaining contigs. The initial genome assembly was further refined using the Pacbio RS_Resequencing.1 module with Quiver consensus calling. The final, circular genome consists of 5,726,633 bp and an overall GC content of 42.0%.

The complete *B. dorei* genome from sample 439 metageomic DNA was closed in the same manner as described above for the 105 sample. The 439 closed genome was significantly smaller than the 105 genome with 5,243,219 bp. Genome annotation for both genomes was done by using the NCBI Prokaryotic Genome Annotation pipeline (Angiuoli et al., 2008), which relies on GeneMarkS+ for gene prediction (Besemer et al., 2001). The NCBI accession numbers for the closed 105 *B. dorei* genome and the 439 *B. dorei* genome are CP007619 and CP008741, respectively. Two-way average nucleotide identities between pairs of genomes were determined as described by Goris et al. (2007).

Methylation patterns obtained from the PacBio data were recovered from both genomes using the assembled genomes as a reference. Methylation data was extracted from the full set of sequence data using Kinetics Tools. This tool utilizes the P_ModificationDetection module in SMRT Portal, which is utilized by the RS Modification Detection as well as the RS Modifications and Motif Detection protocol. The Motif Detection protocol generated motifs by comparing methylation patterns to genomic context.

## Results

PacBio sequencing and assembly from 105 and 439 metagenomic DNA resulted in two closed *Bacteroides dorei* genomes of 5,726,633 and 5,243,219 bp, respectively, The 105 and 439 genomes assembled to an average coverage across each genome of 306.72- and 249.47-fold, respectively. The 16S rRNA and whole genome average nucleotide identities were compared between the 105 and 439 genomes as well as two reference genomes, the nearly complete genome of *B. dorei* DSM 17855 and the complete genome of *B. vulgatus* 8482 (Tables 1, 2). These data show that the 105 and 439 genomes are far more closely related to *B. dorei* than *B. vulgatus*.

**Table 1:** **Percent two-way average nucleotide identities (ANI) at the whole genome level between the *B. dorei* 105 and 439 genomes described here and the nearly complete genome of *B. dorei* DSM 17855 and the complete genome of *B. vulgatus* ATCC 8482**.

**Strain**	**105**	**439**	**17855**	**8422**
105	100	99.50	99.42	96.25
439		100	99.55	96.33
17855			100	96.27
8422				100

**Table 2:** **Percent 16S rRNA identities between the *B. dorei* 105 and 439 genomes described here and the nearly complete genome of *B. dorei* DSM 17855 and the complete genome of *B. vulgatus* ATCC 8482**.

**Strain**	**105**	**439**	**17855**	**8422**
105	100	99.93	100	97.36
439		100	99.93	97.42
17855			100	97.05
8422				100

Methylated GATC motifs were only found in the *B. dorei* from the 105 sample and were completely absent in the *B. dorei* contigs obtained for sample 439 (Table 3). There were 49,007 total methylations detected in *B. dorei* 105, and 38,203 total methylations detected in *B. dorei* 439. Of these, 14,322 and 24,770 methylation sites for the 105 and 439 genomes, respectively, could not be clustered to any motif. In the *B. dorei* genome from the 105 sample, 20,551 GATC methylations and one copy of the DamMT gene were identified. Of 20,554 GATC sites in the 105 genome, only 3 were not detected as methylated. In contrast, the *B. dorei* genome of 5,243,219 bp from sample 439 lacked a DamMT gene and no GATC motif methylations were detected among the 18,908 GATC sites identified.

**Table 3:** **Methylated motif analysis of the 105 and 439 genomes**.

	**Modification type**	**Number of methylations**	**Number of motifs in genome**	**% motifs methylated**	**Number of methylated motifs/kb**	**Number of motifs/kb**	**Mean motif coverage**
**105 MOTIFS**							
GATC	m6A	20,551	20,554	99.99	3.58867	3.58919	147.5
CGMATC	m6A	4353	4353	100.00	0.76013	0.76013	148.8
CAGNNNNNNRTKG	m6A	2948	3196	92.24	0.51479	0.55809	149.3
CAAYNNNNNNCTG	m6A	1808	1812	99.78	0.31572	0.31642	147.1
SAGNNNNNNCTTC	Unknown	1070	1599	66.92	0.18685	0.27922	149.4
GAAGNNNNNNCTS	Unknown	997	1599	62.35	0.17410	0.27922	148.7
CCAYNNNNNNCTG	Unknown	931	1384	67.27	0.16257	0.24168	152.2
GACNNNNNRTTG	m6A	1022	1024	99.80	0.17846	0.17881	147.7
CAAYNNNNNGTC	m6A	1005	1024	98.14	0.17550	0.17881	145.3
Total		34,685	36,545	94.91	6.05679	6.38159	
**439 MOTIFS**							
GAANNNNNNNNNTTC	Unknown	926	5626	16.46	0.17661	1.07300	180.7
GGANNNNNNNNNTCC	m6A	2395	3016	79.41	0.45678	0.57522	177.4
TTCGAA	m6A	2103	2112	99.57	0.40109	0.40281	176.0
GAANNNNNNNNRTGG	Unknown	680	1914	35.53	0.12969	0.36504	178.8
ACAYNNNNNNNTCC	Unknown	922	1367	67.45	0.17585	0.26072	175.8
GGANNNNNNNRTGT	Unknown	777	1367	56.84	0.14819	0.26072	178.1
CCAYNNNNNNNNTCC	m6A	1177	1324	88.90	0.22448	0.25252	174.4
GGANNNNNNNNRTGG	m6A	1136	1324	85.80	0.21666	0.25252	175.2
GAAGNNNNNNNTCC	m6A	643	924	69.59	0.12263	0.17623	174.6
ACAYNNNNNNNTGG	Unknown	536	867	61.82	0.10223	0.16536	177.4
CCANNNNNNNRTGT	Unknown	535	867	61.71	0.10204	0.16536	178.3
CCANNNNNNNCTTC	m6A	630	800	78.75	0.12016	0.15258	178.6
GAAGNNNNNNNTGG	m6A	616	800	77.00	0.11749	0.15258	173.4
GGANNNNNNNCTTCD	Unknown	357	661	54.01	0.06809	0.12607	180.2
Total		13,433	22,969	58.48	2.56198	4.38071	

The number methylation sites observed that could not be clustered to any motif were 14,322 and 24,770 for the 105 and 439 genomes, respectively.

A total of 5,107,008 bp or 89% of the *B. dorei* 105 genome codes directly for ORFs. Of the 20,551 methylated GATC sties found in *B. dorei* 105, 18,816 were found within 3184 of the 4970 annotated open reading frames. Of the 1735 methylated GATC sites identified between ORFs many are likely within promoter regions. Thus, there are 2.80 GATC methylation sites per kbp of non-coding genome space which is slightly lower than the 3.68 GATC methylation sites per kbp of the coding region. This suggests that the evolutionary pressure to maintain GATC sites is stronger in coding regions compared to non-coding regions.

Of the 245 SEED subsystems identified by RAST annotation of the complete *B. dorei* 105 genome, only 13 subsystems had no GATC methylation sites (Table S1). Those include purine utilization, carbon starvation, copper homeostasis, gentisate degradation, salicylate, and gentisate catabolism.

The remaining 232 subsystems were methylated at nearly every GATC site. The Ton and Tol transport systems which provide energy for transport across the outer membrane in Gram negative bacteria was the most highly GATC methylated subsystem, accounting for 6.7% of all GATC methylations and 5.5% of the genome. Transport plays a crucial role in the heterotrophic bacteria that reside in the gut.

Other motifs in both genomes were detected in this analysis as well (Table 3). About 8.6 and 35.2% of all of the motifs detected in the 105 and 439 genomes, respectively, cannot be characterized as to the position of modification on the adenosine ring. The remaining motifs were all methylations of the 6-position of the adenosine ring and less abundant than the GATC modification. Excluding the GATC motif, there are 15,991 motifs remaining in the 105 genome, of which 14,134 (88.4%) are modified. In the 439 genome, there are 22,969 motifs detected, however, only 13,433 (58.5%) are modified. Thus, even non-GATC adenosine methylation sites are much more likely to be methylated in the 105 genome compared to the 439 genome. The phenotypic effects of these methylations are unknown.

As the primary objective of this work was to obtain closed *B. dorei* genomes and examine GATC methylations, the libraries were not prepared in a way that would allow detection of 5-methylcytosine modifications. Both the 105 and 439 genomes each have genes that code for putative DNA cytosine methyltransferases. Hence, we predict that 5′-cytosine methylation is normal in both the 105 and 439 samples.

**Figure 1:**
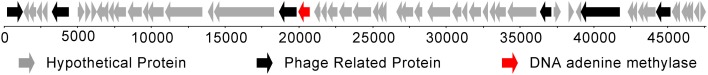
**Location of the DNA adenine methyltransferase (in red) within a bacteriophage present in the Bacteroides dorei genome**.

This is in contrast to the Dam methyltransferase gene, which is only present in the 105 genome. In the 105 genome, a single Dam methyltransferase gene was annotated that is predicted to recognize the GATC motif (Figure 1). This gene is within a bacterial prophage region that is 47,617 bp in length (between bases 1,014,516 and 1,062,132 bp in the genome), making it similar to other bacteriophage orphan DNA methyltransferases found in bacteria (Murphy et al., 2013).

Another interesting observation is that of all of the methylation motifs observed in these two genomes, none is methylated in both genomes. This suggests that the primary source of methyltransferases in these genomes is through lateral transfer, often from phage. Nevertheless, the GATC motif methylation and abundance is the primary difference between the two genomes.

## Discussion

Given that nearly all subsystems can be virtually completely methylated in one strain of a dominant gut bacterium and completely unmethylated in another strain of the same species from a different subject, suggests that epigenetics may be a crucial process regulating the metabolic potential of heterotrophic bacteria in the human gut. The observation that similar species reside in the gut of two individuals is not sufficient to propose that the metabolic capabilities of the two microbiomes are similar. This work suggests that future microbiome studies should consider the methylome when describing the bacterial diversity in the gut. Such analyses are no longer difficult given the latest sequencing technologies.

Although methylation can lead to up-regulation or down-regulation of a gene (Marinus and Casadesus, 2009), it is impossible to say whether the GATC motif affects the expression of *B*. *dorei* genes in the gut. However, the sheer number of GATC methylations found in one of these genomes suggests that GATC methylation is worthy of analysis in future investigations. It is likely that gene expression varies widely between a strain that is fully GATC methylated vs. another that is not. Unfortunately, unlike 16S rRNA, DamMT genes are not sufficiently conserved at the nucleotide level to allow rapid PCR analyses of this gene in metagenomic samples.

The effect of Dam methylation on gut colonization and survival may be significant. For example, Dam methylation of various transport systems affects many processes including import of nutrients, energy transfer, and antibiotic resistance conferred by antibiotic efflux. All of these are important to gut colonization and survival. A Dam mutant of *Haemophilus influenzae* is more susceptible to many antibiotics including erythromycin, tetracycline, kanamycin, and spectinomycin (Sanchez et al., 1997). In most cases, the *H. influenzae* Dam mutant showed not only a lower minimum inhibitory concentration, but also increased susceptibility to higher concentrations of antibiotics (Zaleski and Piekarowicz, 2004) suggesting that Dam methylation has a role in upregulating expression of efflux pumps and ultimately antibiotic resistance.

GATC methylation has also been implicated in pathogenesis by bacteria. DamMT mutants in several species have shown attenuated pathogenesis (Heithoff et al., 1999; Julio et al., 2001, 2002; Watson et al., 2004; Robinson et al., 2005; Balbontin et al., 2006; Mehling et al., 2007; Kim et al., 2008; Murphy et al., 2008) and as GATC methylation is involved in many fundamental bacterial processes including, but not limited to, replication, DNA repair, transcription, and LPS composition (Marinus and Casadesus, 2009), it is not surprising that DamMT deficient strains would be impaired in their ability to interact with eukaryotic hosts. Of course, given that have only two samples were examined here, no estimate of the effects of gut bacterial GATC methylation on type 1 diabetes autoimmunity outcome can be made.

GATC methylation in the *B. dorei* 105 genome appears to be the result of a single orphan DNA adenine methyltransferase gene embedded within a bacterial prophage of unknown origin. These orphan DamMTases have been reported to protect phage DNA that include the GATC motif from restriction (Kossykh et al., 1995; Miller et al., 2003). That may be the case here as the frequency of GATC motifs within the DamMT-containing bacteriophage region is 1.7 times higher than in the 105 genome overall with 6.09 and 3.59 GATC motifs per kb in the prophage and whole genome, respectively.

These orphan DamMT-containing bacteriophage can also regulate lysogeny by the bacteriophage (Murphy et al., 2008). Until this strain is cultured and a mutant is made that lacks DamMTase activity, the phenotype(s) conferred by this gene will remain unknown.

How GATC methylation affects microbiome function is unknown. But given the many bacterial processes affected by GATC methylation, environmental shaping of bacterial genomes through environmental exposures may influence the role of the microbiome in gut diseases. The surprising aspect of this work is that a single dominant species in the gut of these two subjects, *B. dorei*, can vary so radically in methylation patterns simply because of the absence of the DamMT gene in *B. dorei* in one of these subjects. Current and future metagenomic datasets from the gut microbiome should be mined for DamMT genes and GATC methylation sites to determine their abundance and taxonomic breadth.

### Conflict of interest statement

The authors declare that the research was conducted in the absence of any commercial or financial relationships that could be construed as a potential conflict of interest.

## Supplementary material

Table S1 **This is an excel file showing the number of GATC and total methylations in the subsystems identified in the 105 genome**.
